# Supplementary material for: Female mice exhibit resistance to disease progression despite early pathology in a transgenic mouse model inoculated with alpha-synuclein fibrils
Source: Commun Biol. 2025 Feb 22;8:288. doi: 10.1038/s42003-025-07680-1 (PMC11846974; doi:10.1038/s42003-025-07680-1)
Supplement: Supplementary file 1 — Supplementary Information [file 42003_2025_7680_MOESM1_ESM.pdf]

Female mice exhibit resistance to disease progression despite early pathology in a transgenic mouse model inoculated with alpha-synuclein fibrils

Authors: Stephanie Tullo[1,2], Janice Park[2], Daniel Gallino[2], Megan Park[2], Kristie Mar[2], Vladislav Novikov[3], Rodrigo Sandoval Contreras[3], Raihaan Patel[2,4], Esther del Cid-Pellitero[5], Edward A. Fon[5], Wen Luo[6], Irina Shlaifer[6], Thomas M. Durcan[6], Marco A.M. Prado[3,7,8], Vania F. Prado[3,7,8], Gabriel A. Devenyi[2,9], M. Mallar Chakravarty[1,2,4,9]

Affiliations:
[1] Integrated Program in Neuroscience, McGill University, Montreal, Quebec, Canada
[2] Cerebral Imaging Center, Douglas Research Center, McGill University, Verdun, Quebec, Canada
[3]Robarts Research Institute, Schulich School of Medicine, The University of Western Ontario, Ontario, Canada
[4] Department of Biological & Biomedical Engineering, McGill University, Montreal, Quebec, Canada
[5] Department of Neurology and Neurosurgery, Montreal Neurological Institute, McGill University, Montreal, Quebec, Canada
[6] Early Drug Discovery Unit, Montreal Neurological Institute, McGill University, Montreal, Quebec, Canada
[7] Department of Physiology and Pharmacology, Schulich School of Medicine, The University of Western Ontario, Ontario, Canada
[8] Department of Anatomy & Cell Biology, Schulich School of Medicine, The University of Western Ontario, Ontario, Canada
[9] Department of Psychiatry, McGill University, Montreal, Quebec, Canada

*Stephanie Tullo, M. Mallar Chakravarty. Cerebral Imaging Center, Douglas Research Center, Verdun, Canada, H4H 1R3. 514-761-6131 ext. 4781. Email: stephanie.tullo@mail.mcgill.ca, mallar.chakravarty@mcgill.ca.

-7 & 30 dpi					90 dpi					120 dpi			
	**PBS**	**Hu-PFF**	**Total**			**PBS**	**Hu-PFF**	**Total**			**PBS**	**Hu-PFF**	**Total**
**M**	30	34	**64**		**M**	21	23	**44**		**M**	9	5	**14**
**F**	31	33	**64**		**F**	23	20	**43**		**F**	9	8	**17**
			**128**					**87**					**31**

Supplementary Table 1. Number of mice per time point for MRI and behavioural testing. Phosphate buffered saline (PBS); human aSyn preformed fibrils (Hu-PFF); male (M); female (F).

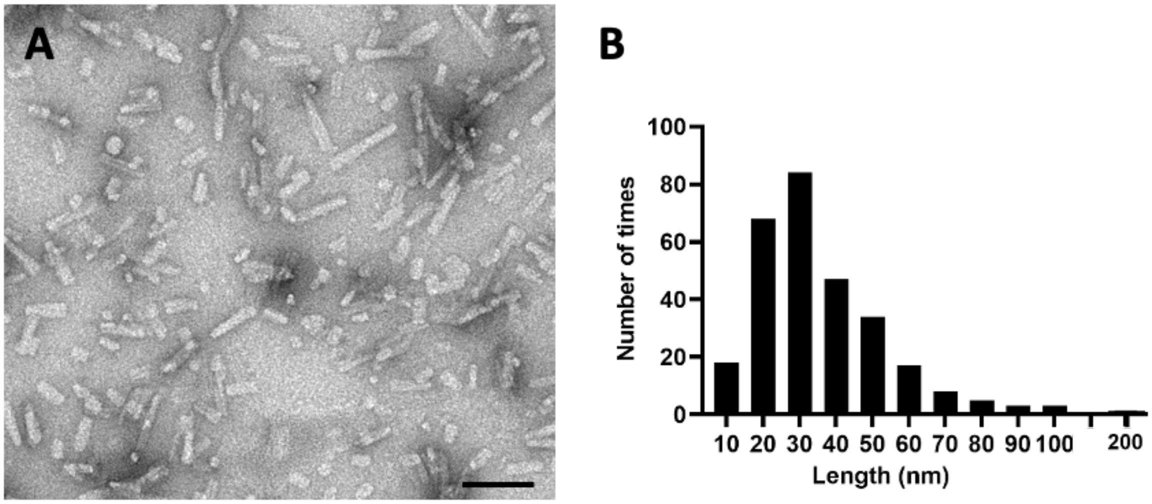

Supplementary Figure 1. Human alpha-synuclein preformed fibrils (Hu-PFF) characterization. [A] Representative photomicrographs of Hu-PFF staining by negative staining and visualized using Tecnai G2 Spirit electron microscope. [B] Histograms showed the Hu-PFF length distribution measured using ImageJ software and their distribution plotted using GraphPad Prism software. Human syn-PFFs sonicated for 30 seconds (n= 288, length average= 35.76nm, median length= 31.65nm, minimal length = 9nm, maximal length= 200 nm).

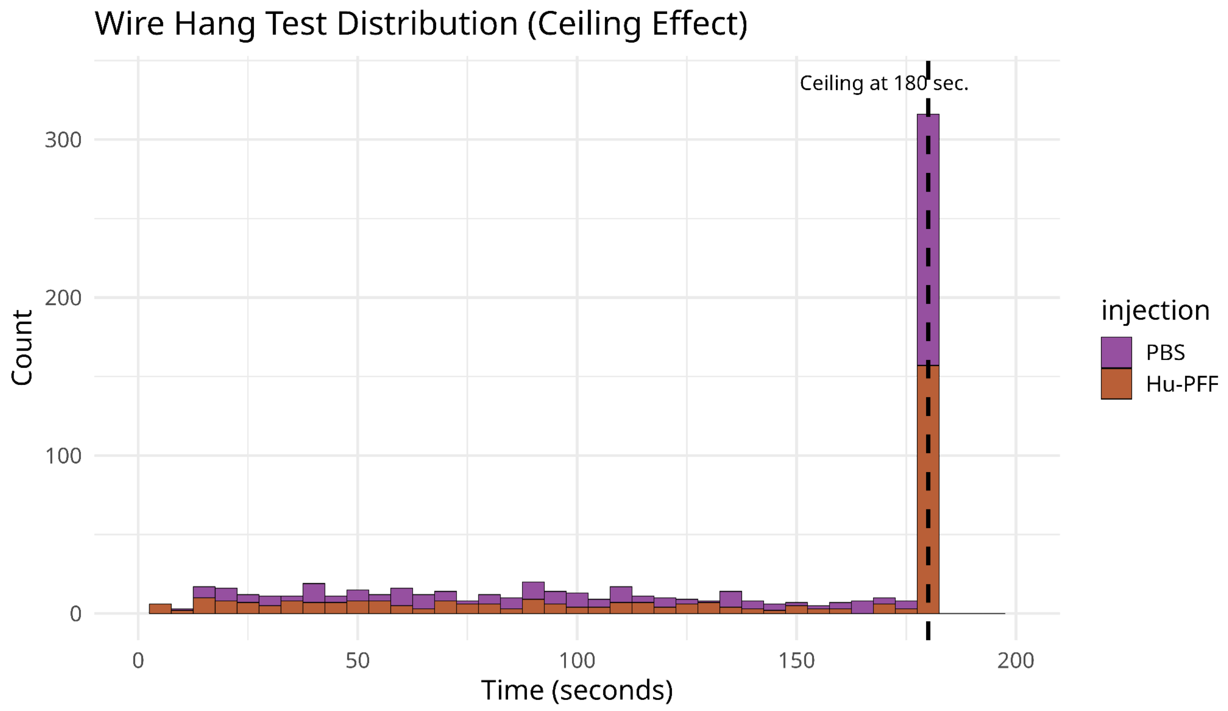

Supplementary Figure 2. Data distribution for wire hang test highlights ceiling effect. This histogram highlights the spread of the data (n=716) such that there is a ceiling effect in the wire hang test, where many subjects reached the maximum allotted time for wire hang and accordingly successfully completed the test, as any latency under the 180 seconds is deemed a failed attempt. Using the Cox Proportional Hazard model, this framework provides a more accurate and robust way to analyze and interpret this type of data. This method captures both the time taken and the success/failure rates, offering a nuanced picture of performance across groups. PBS-injected mice are in purple and Hu-PFF mice are in orange. Histogram binning was performed at 5.

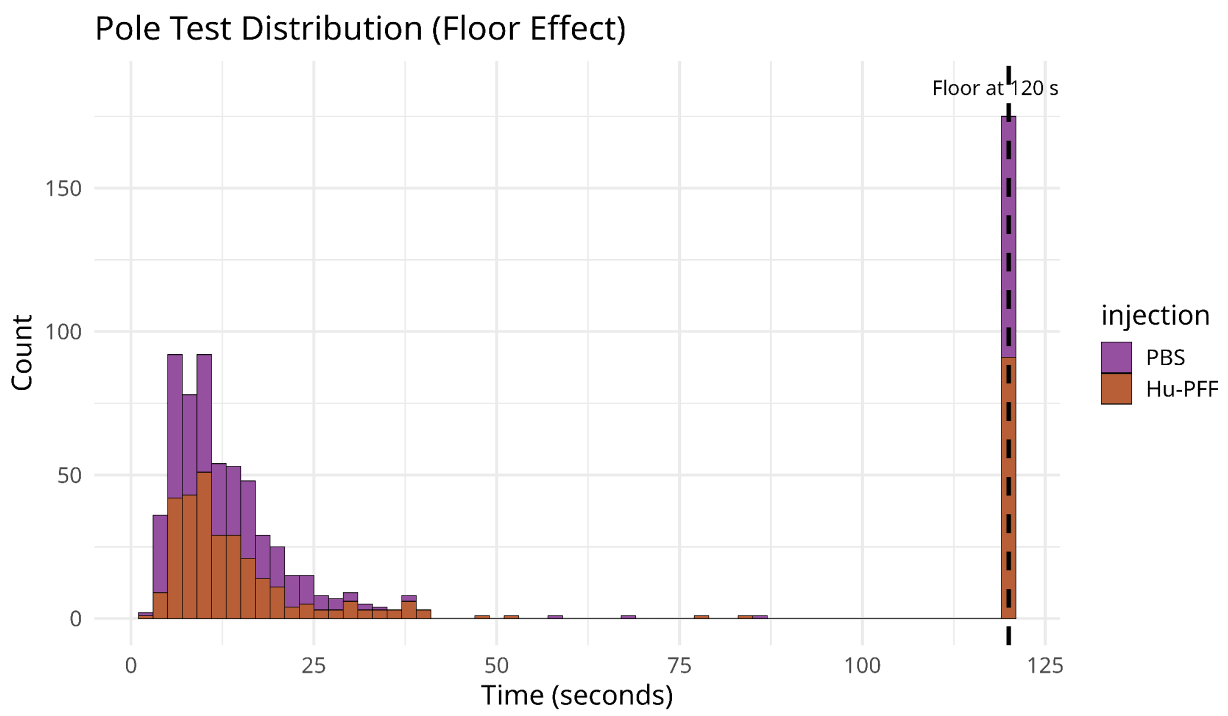

Supplementary Figure 3. Data distribution for pole test highlights floor effect. This histogram highlights the spread of the data (n=768) such that there is a floor effect in the pole test, where many subjects failed to perform the task and consequently were allotted the maximum time (120 seconds). Using the Cox Proportional Hazard model, this framework provides a more accurate and robust way to analyze and interpret this type of data. This method captures both the time taken and the success/failure rates, offering a nuanced picture of performance across groups. PBS-injected mice are in purple and Hu-PFF mice are in orange. Histogram binning was performed at 2.

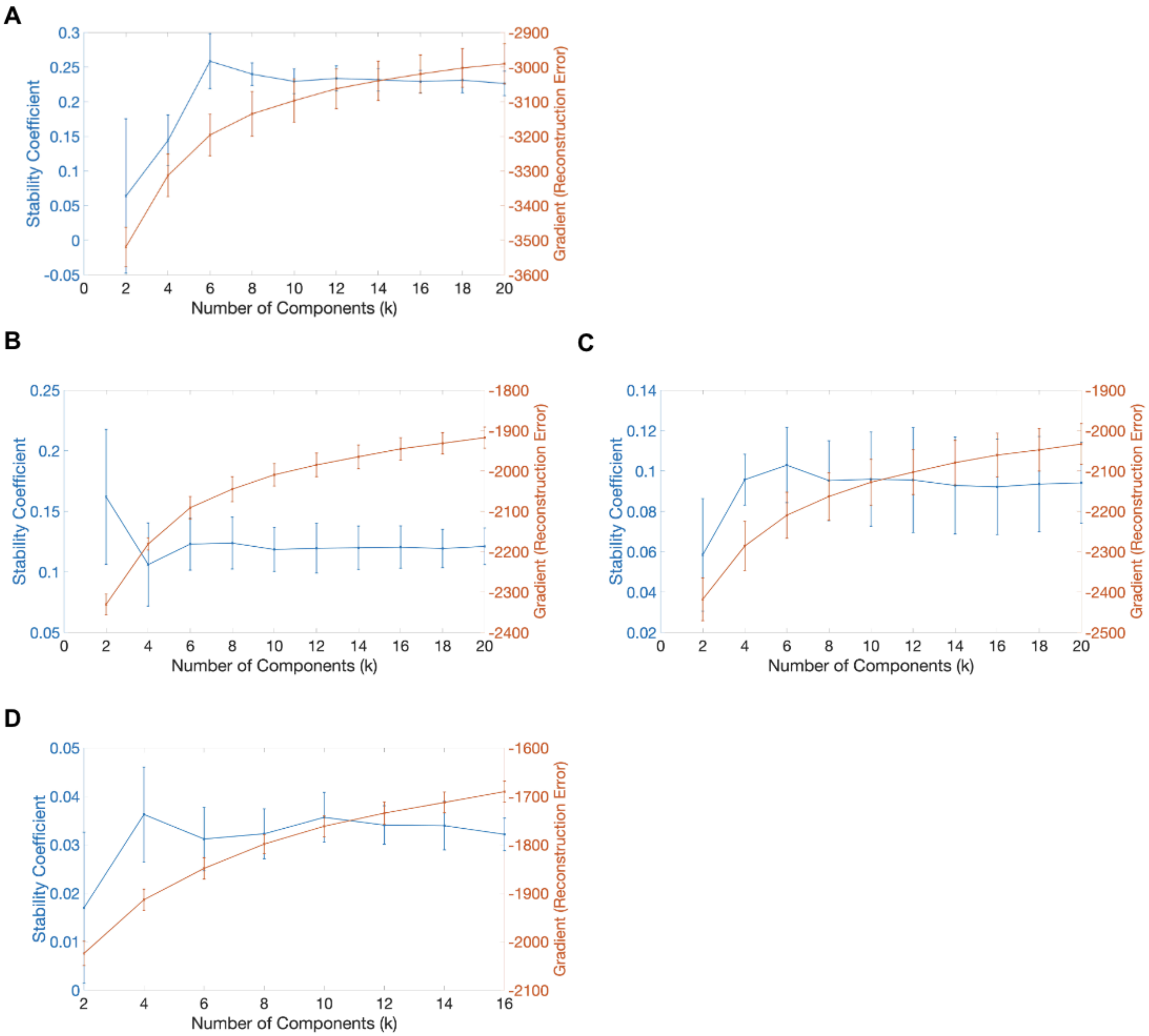

Supplementary Figure 4. Stability analysis for k=3 to 10 OPNMF component runs. [A-C] Accuracy (stability coefficient; blue) and gradient reconstruction error (red) measures at 90 dpi for [A] all subjects, [B] males and [C] female mice respectively. [D] Accuracy and gradient reconstruction error measures for 120 dpi OPNMF run. Gradient in reconstruction error (red) is the quantification of the gain in accuracy provided by increasing the number of components from one granularity to the next. The stability of a decomposition (blue) is measured by assessing the similarity of output spatial components across varying splits of subjects; 5 splits were performed at each granularity. For all 4 OPNMF runs, k=6 components were chosen based on the criteria of choosing the highest stability measure with the biggest gain in accuracy (reconstruction error). The error bars represent the standard deviation of the stability coefficient (blue) or the reconstruction error (red).

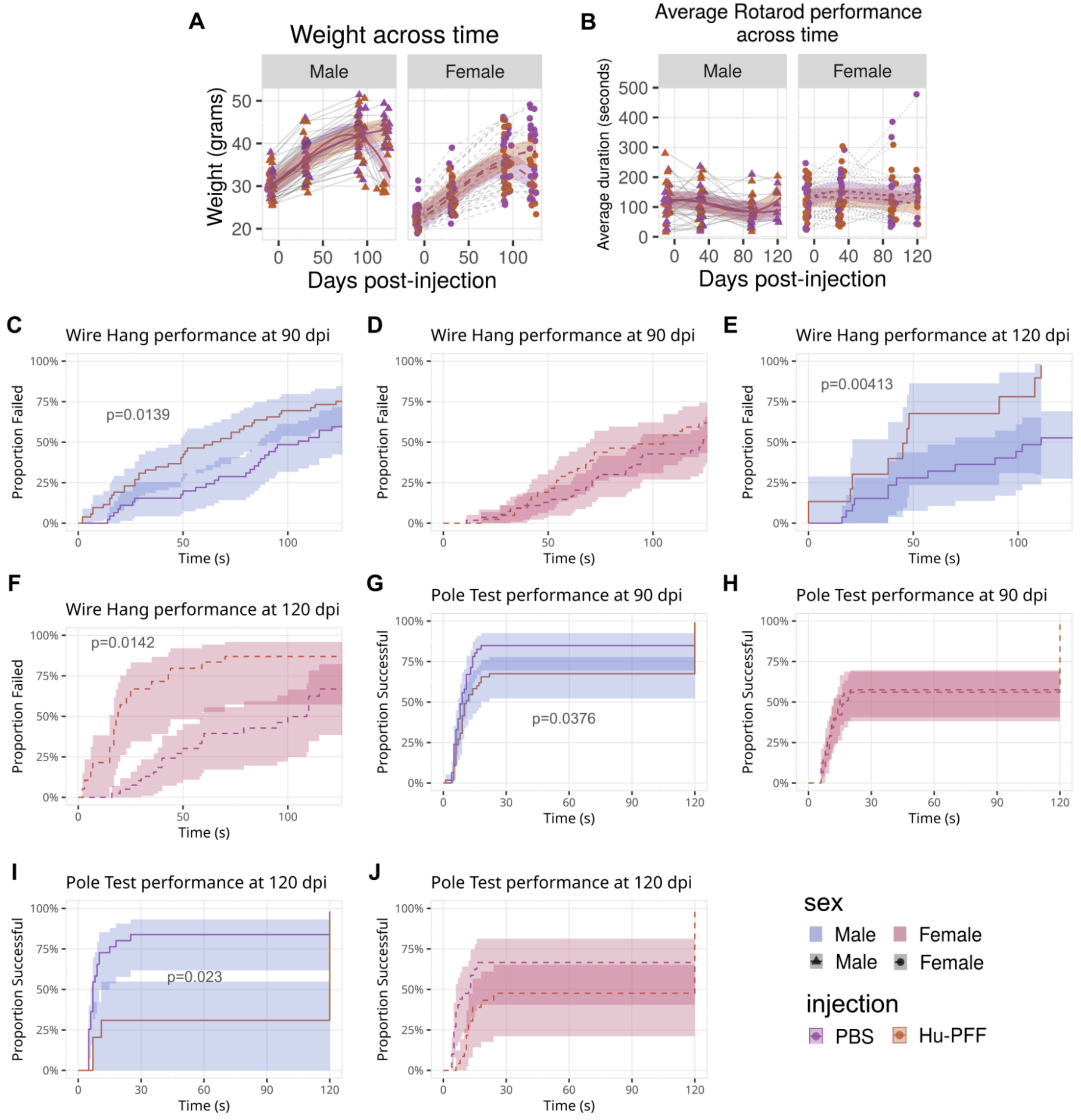

Supplementary Figure 5. Sex differences in motor symptomatology and performance on motor tasks. [A] Weight trend across disease progression. Significant inverted U-shaped trajectory for Hu-PFF-injected mice, with weight loss as of 90 dpi (compared to PBS-injected mice) (n=329; p=0.0056). No sex-specific differences were observed when examining the triple interaction between group, sex, and time (days post-injection). Shading represents ±1 standard error of the mean. [B] Average rotarod performance across time showed no significant differences between injection groups and sex (n=256). Shading represents ±1 standard error of the mean. [C,F] Sex-specific wire-hang performance at 90 and 120 dpi. Significant difference in the proportion of mice that failed (<3 minutes) between injections groups for male mice (90 dpi:

n=96; p=0.0.139; 120 dpi: n=42; p=0.00413), with higher rates of failure for the Hu-PFF-injected mice (red dashed line) and injection groups differences for female mice at 120 dpi (90 dpi: n=90; p>0.05; 120 dpi: n=51; p=0.0142). [G,J] Sex-specific pole test performance at 90 and 120 dpi. Male Hu-PFF-injected mice had lower proportions of mice successfully passing the test, and took significantly longer to descend the pole compared to their saline injected counterparts at both time points (90 dpi: n=99; p=0.0376; 120 dpi: n=36; p=0.023). No statistical difference between injection for female mice was observed at 90 (n=90) and 120 dpi (n=51). Shading represents a 95% confidence interval. Purple colour denotes PBS-injected mice and orange colour denotes Hu-PFF injected mice. Line type was used to denote each of the sexes: solid line (with blue shading) for male and dashed line (with red shading) for female mice. Data point shapes also denote the sex of the mice: triangle for males and round for females.

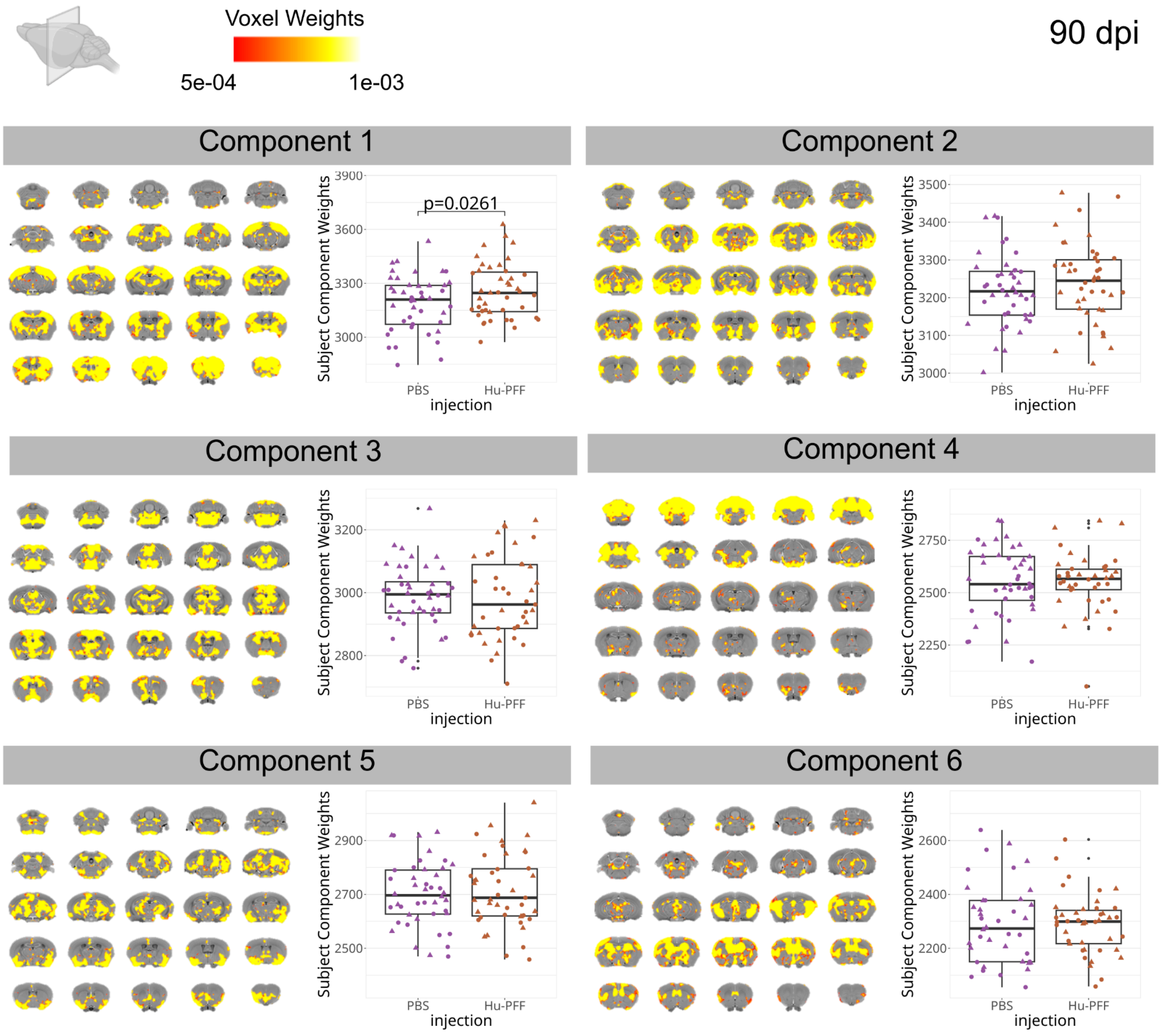

Supplementary Figure 6. Results of the 90 dpi OPNMF run for all 6 components. Coronal slices of a mouse brain average displayed from posterior to anterior slices. Colourmap denotes voxel-wise component weights. For each component, the spatial pattern of voxel component scores plotted onto the average mouse brain, depicting the networks of voxels sharing a similar variance pattern (left) and group differences of subject component weightings, describing how each subject loads onto the identified atrophy pattern were assessed using general linear models (right) for each of the 6 components. Component 1 was the only component where the injection group was significantly associated with OPNMF voxel weights (n=87 mice; p=0.0261). Purple for PBS-injected mice, orange for Hu-PFF-injected mice, triangle points for male and circular points for female mice. The error bars indicate the data spread up to 1.5 times the interquartile range.

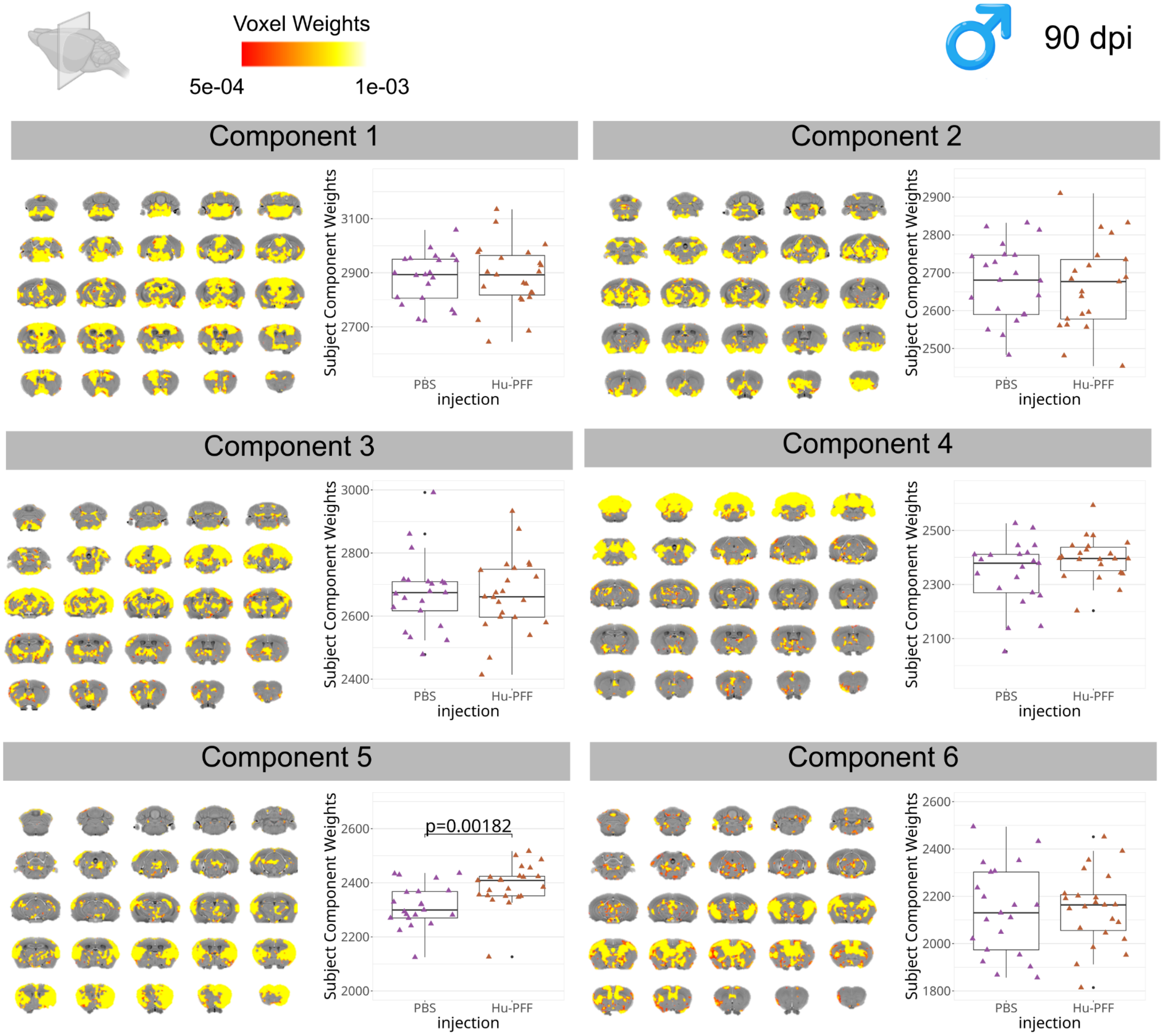

Supplementary Figure 7. Results of the 90 dpi OPNMF run for all 6 components for M83 Hu-PFF- and PBS-injected male mice. Coronal slices of a mouse brain average displayed from posterior to anterior slices. Colourmap denotes voxel-wise component weights. For each component, the spatial pattern of voxel component scores plotted onto the average mouse brain, depicting the networks of voxels sharing a similar variance pattern (left) and group differences of subject component weightings, describing how each subject loads onto the identified atrophy pattern were assessed using general linear models (right) for each of the 6 components. Component 5 was the only component where the injection group was significantly associated with OPNMF voxel weights (n=44 male mice; p=0.00182). Purple for PBS-injected mice, orange for Hu-PFF-injected mice, triangle points for male and circular points for female mice. The error bars indicate the data spread up to 1.5 times the interquartile range.

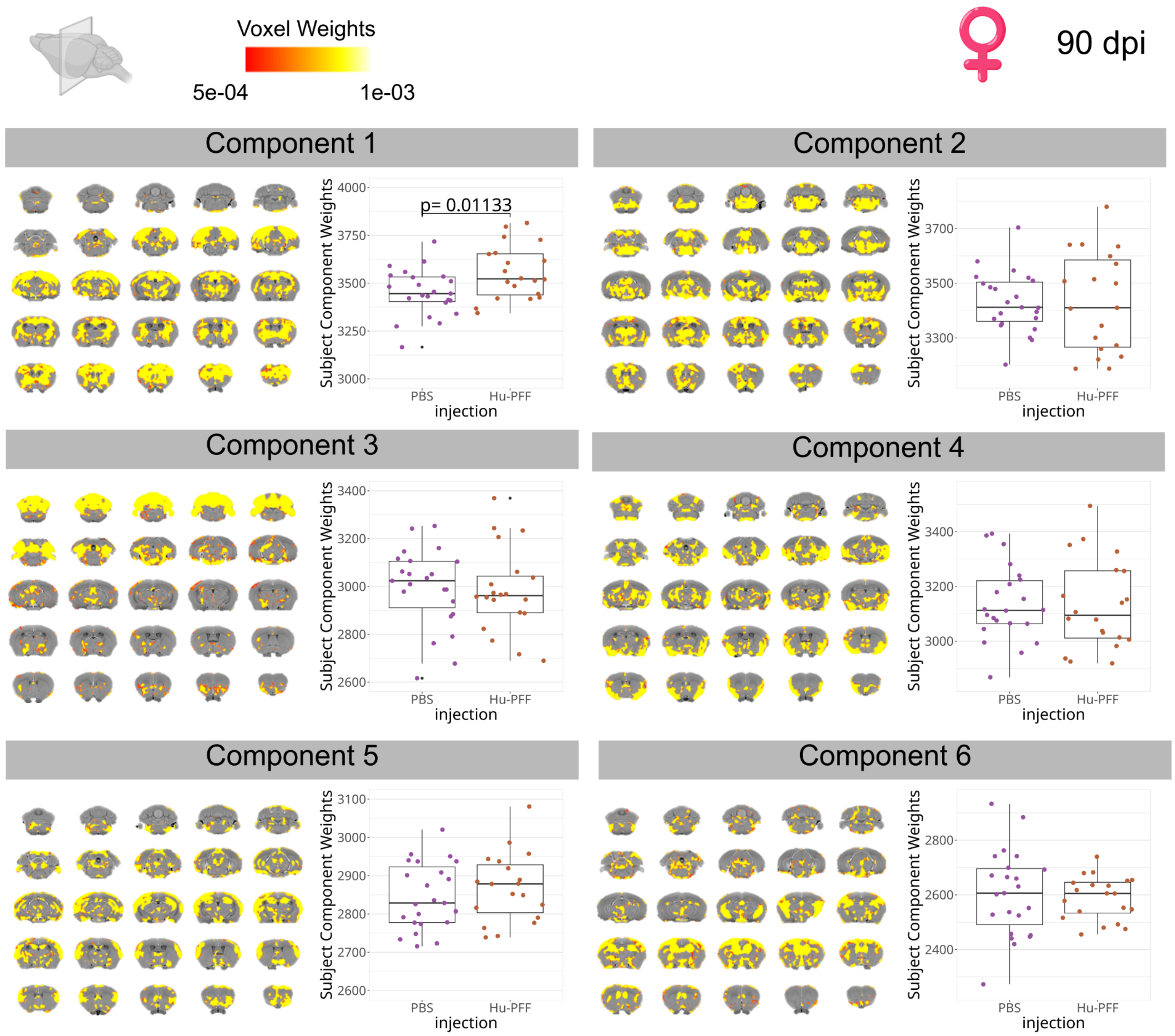

Supplementary Figure 8. Results of the 90 dpi OPNMF run for all 6 components for M83 Hu-PFF- and PBS-injected female mice. Coronal slices of a mouse brain average displayed from posterior to anterior slices. Colourmap denotes voxel-wise component weights. For each component, the spatial pattern of voxel component scores plotted onto the average mouse brain, depicting the networks of voxels sharing a similar variance pattern (left) and group differences of subject component weightings, describing how each subject loads onto the identified atrophy pattern were assessed using general linear models (right) for each of the 6 components. Component 1 was the only component where the injection group was significantly associated with OPNMF voxel weights (n=43 female mice; p=0.0113). Purple for PBS-injected mice, orange for Hu-PFF-injected mice, triangle points for male and circular points for female mice. The error bars indicate the data spread up to 1.5 times the interquartile range.

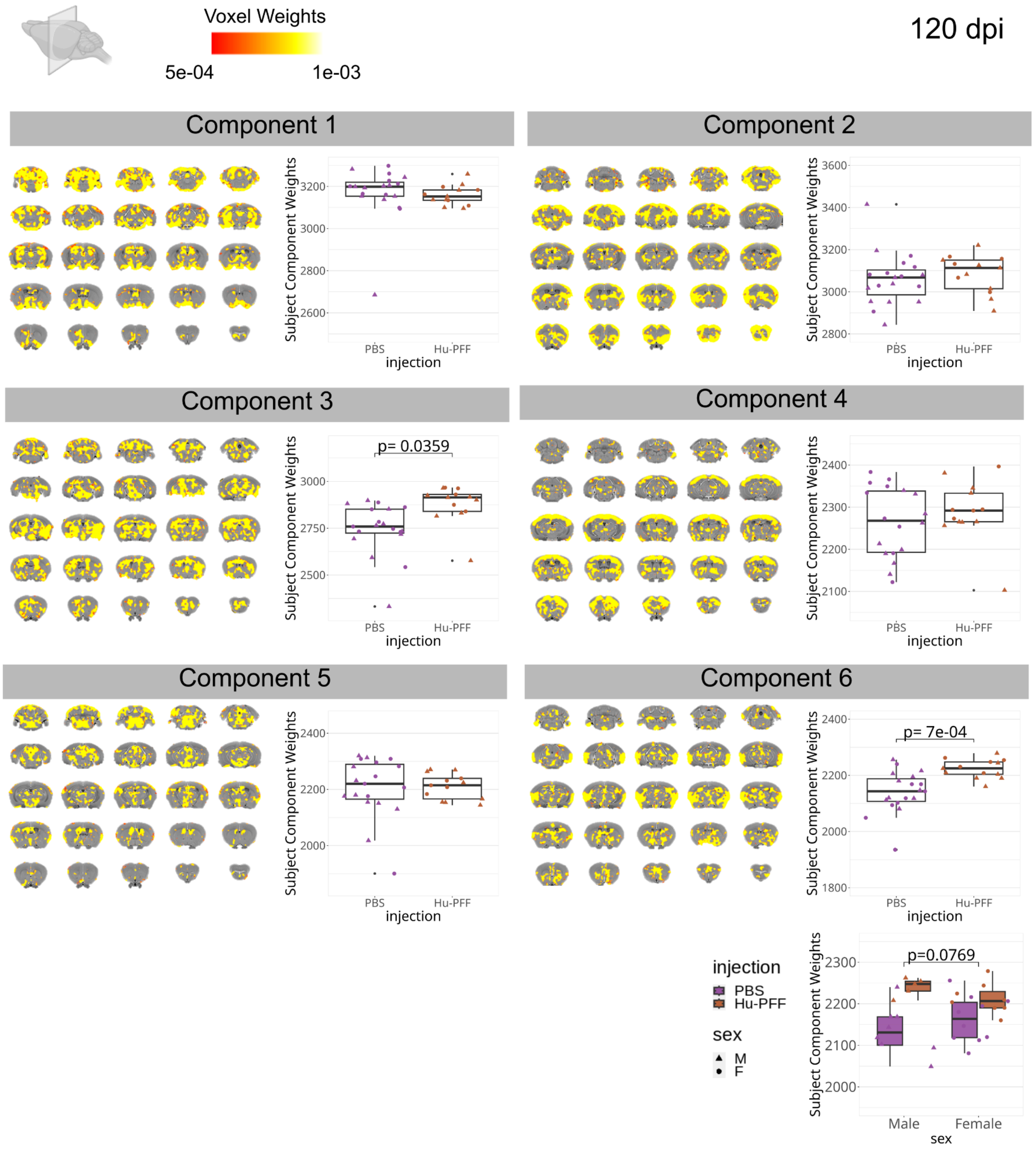

Supplementary Figure 9. Results of the 120 dpi OPNMF run for all 6 components. Coronal slices of a mouse brain average displayed from posterior to anterior slices. Colour map denotes voxel-wise component weights. For each component, the spatial pattern of voxel component scores plotted onto the average mouse brain, depicting the networks of voxels sharing a similar variance pattern (left) and group differences of subject component weightings, describing how each subject loads onto the identified atrophy pattern were assessed using general linear models (right) for each of the 6 components. For components 3 and 6, the injection group was

significantly associated with OPNMF voxel weights (n=32 mice; component 3: p=0.0359; component 6: p=0.0007). Component 3 consists of largely the entire bilateral thalamic nuclei, cerebellum, and brainstem regions, as well as some hypothalamic areas while component 6 consists of mainly subcortical regions such as the striatum, pallidum, lateral areas of the hippocampus, as well as midbrain and the lateral ventricles. For component 6, we also observe a trend-level marginally significant injection group by sex interaction (p=0.0769), where we observe a trend level effect of higher subject weights for male Hu-PFF-injected mice (orange triangular markers). Although sex-specific covariance patterns were generated for the 90 dpi time point, given the high numbers of attrition due to disease progression (with <8 Hu-PFF male mice remaining), there weren't enough mice to perform such analysis at this 120 dpi time point. Purple for PBS-injected mice, orange for Hu-PFF-injected mice, triangle points for male and circular points for female mice. The error bars indicate the data spread up to 1.5 times the interquartile range.

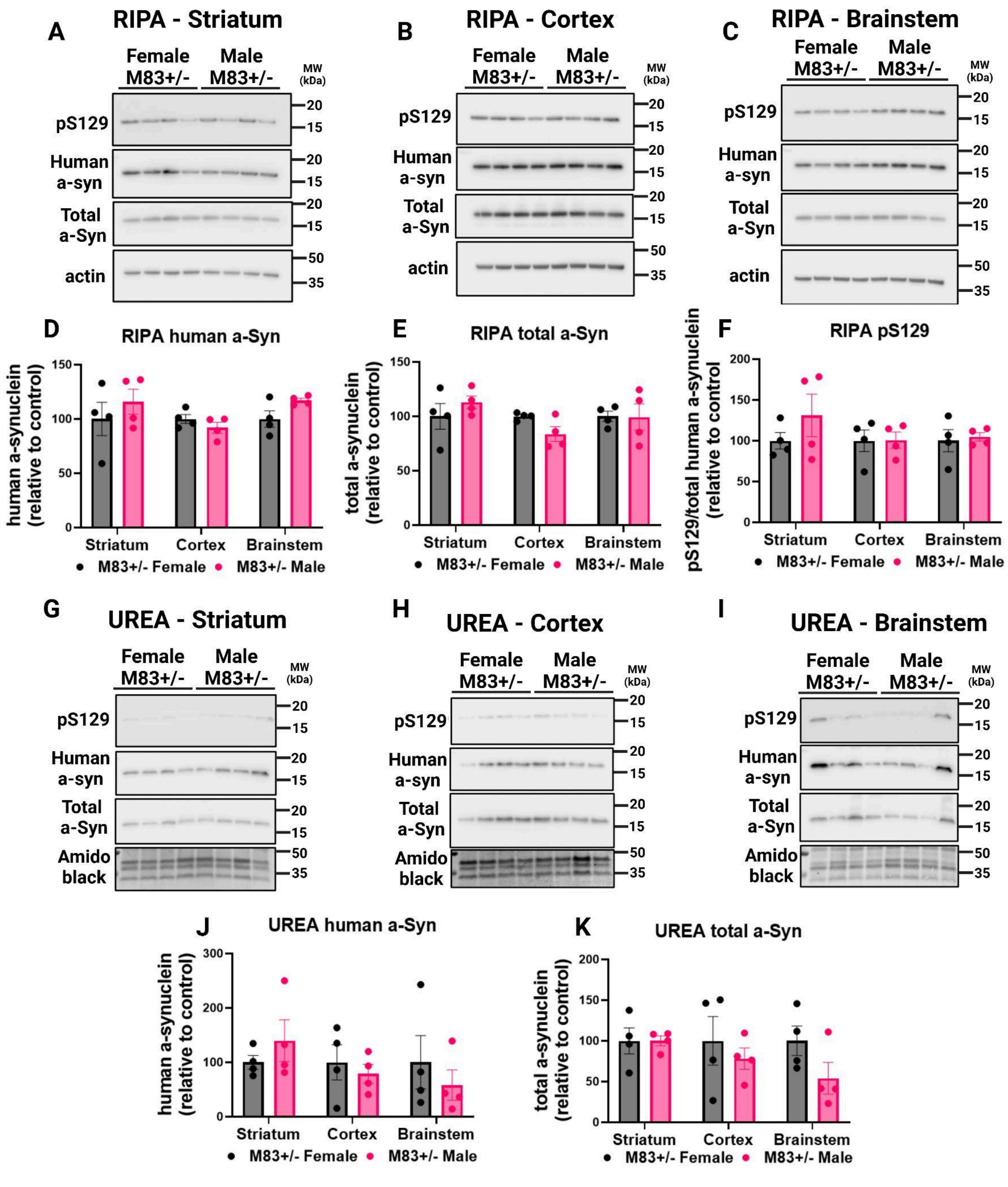

Supplementary Figure 10. Results of the Western Blotting examining sex-differences. (A-C) RIPA fraction results for striatum, cortex, and brainstem tissues, respectively (n= 8 mice; 4 males and 4 females). Human, mouse, and phosho129 a-synuclein levels were quantified and analyzed using unpaired t-tests. (D-F) Insoluble fractions obtained (UREA) were run and quantified similarly. No significant differences were observed for either fractions and signals. Error bars represent standard error of the mean.

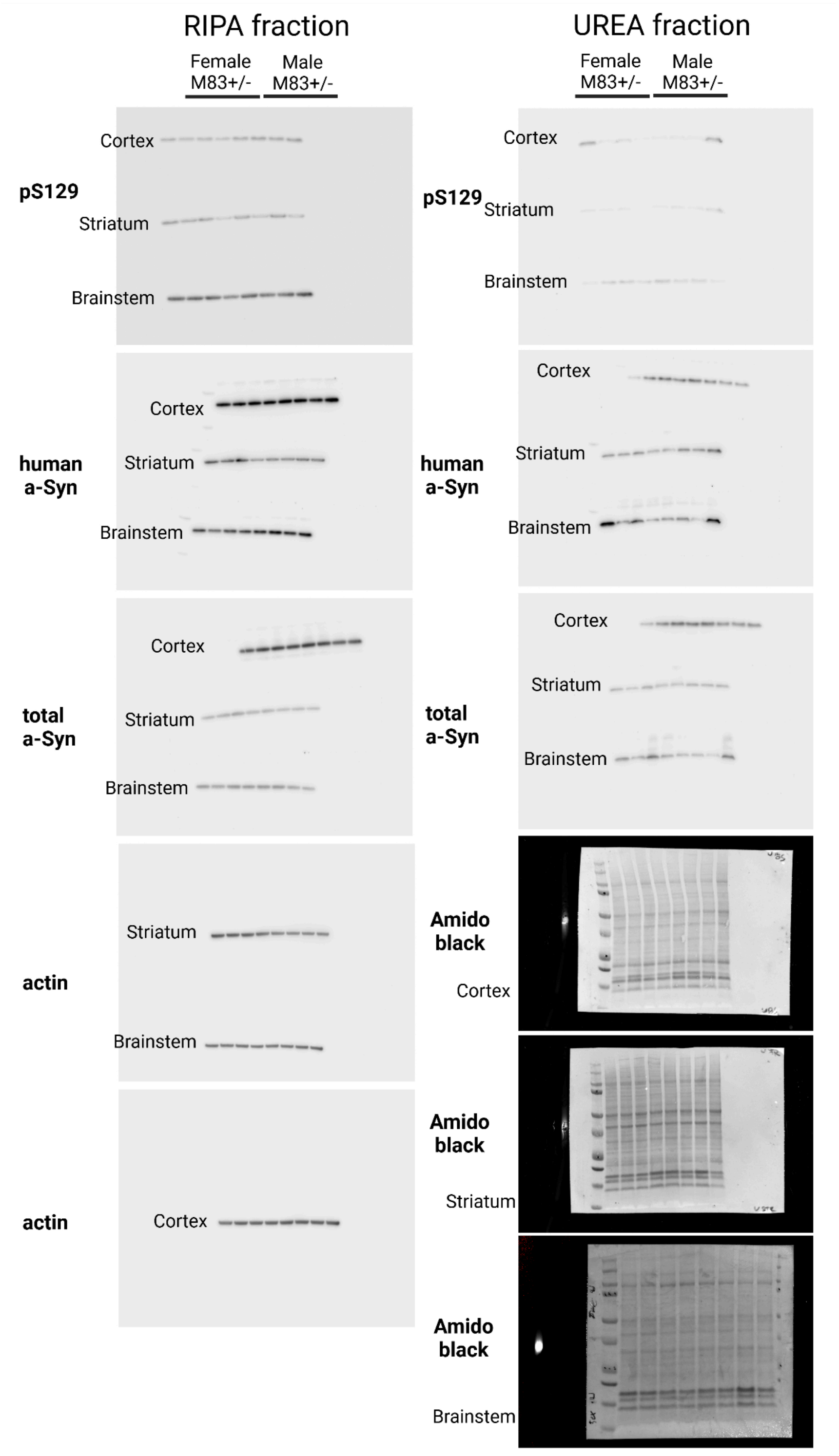

Supplementary Figure 11. Uncropped and unedited western blot images. (left) RIPA fraction results for cortex, striatum and brainstem tissues, respectively (n= 8 mice; 4 males and 4 females). (right) Insoluble fractions obtained (UREA) were run and quantified similarly. No significant differences were observed for either fractions and signals.
