# Supplementary material for: Female mice exhibit resistance to disease progression despite early pathology in a transgenic mouse model inoculated with alpha-synuclein fibrils
Source: Commun Biol. 2025 Feb 22;8:288. doi: 10.1038/s42003-025-07680-1 (PMC11846974; doi:10.1038/s42003-025-07680-1)
Supplement: Supplementary file 2 — Reporting Summary [file 42003_2025_7680_MOESM2_ESM.pdf]

nature portfolio

Corresponding author(s): Stephanie Tullo

Last updated by author(s): Jan 6, 2025

Reporting Summary

Nature Portfolio wishes to improve the reproducibility of the work that we publish. This form provides structure for consistency and transparency in reporting. For further information on Nature Portfolio policies, see our Editorial Policies and the Editorial Policy Checklist.

Statistics

For all statistical analyses, confirm that the following items are present in the figure legend, table legend, main text, or Methods section.

n/a	Confirmed	
☐	☒	The exact sample size (*n*) for each experimental group/condition, given as a discrete number and unit of measurement
☐	☒	A statement on whether measurements were taken from distinct samples or whether the same sample was measured repeatedly
☐	☒	The statistical test(s) used AND whether they are one- or two-sided<br>*Only common tests should be described solely by name; describe more complex techniques in the Methods section.*
☐	☒	A description of all covariates tested
☐	☒	A description of any assumptions or corrections, such as tests of normality and adjustment for multiple comparisons
☐	☒	A full description of the statistical parameters including central tendency (e.g. means) or other basic estimates (e.g. regression coefficient) AND variation (e.g. standard deviation) or associated estimates of uncertainty (e.g. confidence intervals)
☐	☒	For null hypothesis testing, the test statistic (e.g. *F*, *t*, *r*) with confidence intervals, effect sizes, degrees of freedom and *P* value noted<br>*Give P values as exact values whenever suitable.*
☒	☐	For Bayesian analysis, information on the choice of priors and Markov chain Monte Carlo settings
☐	☒	For hierarchical and complex designs, identification of the appropriate level for tests and full reporting of outcomes
☐	☒	Estimates of effect sizes (e.g. Cohen's *d*, Pearson's *r*), indicating how they were calculated

Our web collection on statistics for biologists contains articles on many of the points above.

Software and code

Policy information about availability of computer code

Data collection	No software was used for data collection
Data analysis	All code for MRI processing tecniques (eg: preprocessing, deformation-based morphometry, OPNMF) is available on Github; https://github.com/CoBrALab. Code for analysis is available on Zenodo (doi.org/10.5281/zenodo.14655730).

For manuscripts utilizing custom algorithms or software that are central to the research but not yet described in published literature, software must be made available to editors and reviewers. We strongly encourage code deposition in a community repository (e.g. GitHub). See the Nature Portfolio guidelines for submitting code & software for further information.

Data

Policy information about availability of data

All manuscripts must include a data availability statement. This statement should provide the following information, where applicable:

- Accession codes, unique identifiers, or web links for publicly available datasets
- A description of any restrictions on data availability
- For clinical datasets or third party data, please ensure that the statement adheres to our policy

All source data, MRI outputs and code have been made available in an open repository (doi.org/10.5281/zenodo.14655730).

Research involving human participants, their data, or biological material

Policy information about studies with human participants or human data. See also policy information about sex, gender (identity/presentation), and sexual orientation and race, ethnicity and racism.

Reporting on sex and gender	The focus of this paper is examining sex-specific differences in the disease progression (survival, neuroanatomy, symptomatology, etc.). Given the use of both male and female mice here, all analyses are sex focused; based on the biology of the mice, and in the same vein, no gender analyses were conducted.
Reporting on race, ethnicity, or other socially relevant groupings	not applicable
Population characteristics	not applicable
Recruitment	not applicable
Ethics oversight	All study procedures were performed in accordance with the Canadian Council on Animal Care and approved by the McGill University Animal Care Committee (AUP 2018-8068), and the University of Western Ontario (2020-162; 2020-163).

Note that full information on the approval of the study protocol must also be provided in the manuscript.

Field-specific reporting

Please select the one below that is the best fit for your research. If you are not sure, read the appropriate sections before making your selection.

☒ Life sciences ☐ Behavioural & social sciences ☐ Ecological, evolutionary & environmental sciences

For a reference copy of the document with all sections, see nature.com/documents/nr-reporting-summary-flat.pdf

Life sciences study design

All studies must disclose on these points even when the disclosure is negative.

Sample size	Sample sizes were chosen based on previous work by our group and other groups conducting longitudinal MRI analyses in mouse models of neurodegenerative diseases.
Data exclusions	No data were excluded
Replication	The findings reported here replicate previous findings from our group (Tullo et al., 2023) in terms of spatial covariance pattern of alpha-synuclein-induced pathology at 90 days post-injection.
Randomization	Mice were allocated to injection group at random prior to their start in the experiment
Blinding	Investigators were blind to injection group allocation at both stages of data collection and data analysis.

Reporting for specific materials, systems and methods

We require information from authors about some types of materials, experimental systems and methods used in many studies. Here, indicate whether each material, system or method listed is relevant to your study. If you are not sure if a list item applies to your research, read the appropriate section before selecting a response.

Materials & experimental systems

n/a	Involved in the study	
☐	☒	Antibodies
☒	☐	Eukaryotic cell lines
☒	☐	Palaeontology and archaeology
☐	☒	Animals and other organisms
☒	☐	Clinical data
☒	☐	Dual use research of concern
☒	☐	Plants

Methods

n/a	Involved in the study	
☒	☐	ChIP-seq
☒	☐	Flow cytometry
☐	☒	MRI-based neuroimaging

Antibodies

Antibodies used	The following primary antibodies were used: phospho S129 (1:1000, Cat# ab51253, Abcam, RRID:AB_869973), anti-human a-Syn (1:1000, Cat# ab27766, Abcam, RRID:AB_727020), anti-alpha synuclein (1:1000, Cat# 610787, BD Biosciences, RRID: AB_398108), anti-actin HRP (1:25,000, Cat#A3854, Sigma-Aldrich, RRID:AB_262011).
Validation	*Describe the validation of each primary antibody for the species and application, noting any validation statements on the manufacturer's website, relevant citations, antibody profiles in online databases, or data provided in the manuscript.*

Animals and other research organisms

Policy information about studies involving animals; ARRIVE guidelines recommended for reporting animal research, and Sex and Gender in Research

Laboratory animals	Transgenic hemizygous M83 mice (B6; C3H-Tg[SNCA]83Vle/J) bred in-house (F4-6), expressing one copy of human alpha-synuclein bearing the familial PD-related A53T mutation under the control of the mouse prion protein promoter (TgM83+/-) (Giasson et al., 2002), in addition to the endogenous mouse alpha-synuclein, maintained on a C57BL/C3H background. Mice were 10 weeks old at the start of the experiment.
Wild animals	not applicable
Reporting on sex	The focus of this paper is examining sex-specific differences in the disease progression (survival, neuroanatomy, symptomatology. etc.). Both male and fermale mice were used and each analysis examined the interaction of sex and the inoculum the mice received.
Field-collected samples	not applicable
Ethics oversight	All study procedures were performed in accordance with the Canadian Council on Animal Care and approved by the McGill University Animal Care Committee (AUP 2018-8068), and the University of Western Ontario (2020-162; 2020-163).

Note that full information on the approval of the study protocol must also be provided in the manuscript.

Plants

Seed stocks	not applicable
Novel plant genotypes	not applicable
Authentication	not applicable

Magnetic resonance imaging

Experimental design

Design type	Structual T1w imaging
Design specifications	Mice were scanned at -7, 30, 90 and 120 days post-injection.
Behavioral performance measures	Three motor tasks were administered; pole test, rotarod and wire hang. Duration for each task was recorded. For the pole test and wire hang, whether the mice performed the task correctly (failed/succeded) was also examined using cox proportional hazard modelling.

Acquisition

Imaging type(s)	Structual imaging
Field strength	7 Tesla
Sequence & imaging parameters	FLASH (Fast Low Angle SHot) were acquired for each subject at each time point (TE/TR of 4.5 ms/20 ms, 100 µm isotropic voxels, 2 averages, scan time= 14 min, flip angle=20°)
Area of acquisition	whole brain

Diffusion MRI ☐ Used ☒ Not used

Preprocessing

Preprocessing software	https://github.com/CobraLab/documentation/wiki/Mouse-Scan-Preprocessing
Normalization	Intensity inhomogeneity was corrected using N4ITK (Tustison et al., 2010) at a minimum spline distance of 5 mm
Normalization template	Data was not normalized to a template; all analyses were performed in subject space
Noise and artifact removal	denoised using patch-based adaptive non-local means algorithm (Coupé et al., 2008)
Volume censoring	*Define your software and/or method and criteria for volume censoring, and state the extent of such censoring.*

Statistical modeling & inference

Model type and settings	Univariate analysis: linear mixed effects models (~injection_group*sex*time point + (1|subject_ID))
Effect(s) tested	Neuroanatomical change over time (on a voxel-basis) was analyzed using linear mixed effects modelling

Specify type of analysis: ☒ Whole brain ☐ ROI-based ☐ Both

Statistic type for inference (See Eklund et al. 2016)	voxel-wise
Correction	not applicable

Models & analysis

n/a	Involved in the study
☒	☐ Functional and/or effective connectivity
☒	☐ Graph analysis
☐	☒ Multivariate modeling or predictive analysis

Multivariate modeling and predictive analysis	Whole brain spatial covariance patterns were assessed using orthogonal projective non-negative matrix factorization; linear models were performed to examine injection-related brain patterns.
